# The Diel Rhythms of Biosonar Behavior in the Yangtze Finless Porpoise (*Neophocaena asiaeorientalis asiaeorientalis*) in the Port of the Yangtze River: The Correlation between Prey Availability and Boat Traffic

**DOI:** 10.1371/journal.pone.0097907

**Published:** 2014-05-13

**Authors:** Zhitao Wang, Tomonari Akamatsu, Kexiong Wang, Ding Wang

**Affiliations:** 1 The Key Laboratory of Aquatic Biodiversity and Conservation of the Chinese Academy of Sciences, Institute of Hydrobiology, Chinese Academy of Sciences, Wuhan, China; 2 University of Chinese Academy of Sciences, Beijing, China; 3 National Research Institute of Fisheries Engineering, Fisheries Research Agency, Ibaraki, Japan; 4 CREST, Japan Science and Technology Agency, Tokyo, Japan; University of Auckland, New Zealand

## Abstract

Information on the habitat use of the critically endangered Yangtze finless porpoise (*Neophocaena asiaeorientalis asiaeorientalis*) is critical for its conservation. The diel biosonar behavior of the porpoise in the port areas of the Yangtze River was examined along with simultaneous observations of fish density and boat traffic. Biosonar pulses from the porpoises were detected for 1233 min (5.77%) over a 21,380 min duration of effective observations. In total, 190 (5.63%) buzzes (an indication of prey capture attempts) were recorded among the 3372 identified click trains. Of the 168 echolocation encounters (bouts of click trains less than eight min apart), 150 (89.3%) involved single animals, indicating that solitary porpoises were frequently present and feeding in the port areas. Significant diel patterns were evident involving the biosonar behavior of the porpoises (including click trains and buzzes), fish density and boat traffic. The frequencies of the click trains and buzzes were significantly lower during the day than in the evening and at night, which suggests that porpoises in this region are primarily engaged in crepuscular and nocturnal foraging. The lack of a significant diel pattern in the echolocation encounters indicates the importance of the port in porpoise conservation. A forced feeding schedule may be associated with the lack of a significant correlation between porpoise acoustics and boat traffic. Overall, prey availability appears to be the primary factor that attracts porpoises. Additionally, porpoises tend to migrate or remain downstream in the morning and migrate or remain upstream in the evening, most likely to follow their prey. The findings of this study can be used to improve the conservation of the Yangtze finless porpoise.

## Introduction

A genetically and morphologically distinct cetacean species, the Yangtze finless porpoise (*Neophocaena asiaeorientalis asiaeorientalis*) is listed as Critically Endangered on the International Union for the Conservation of Nature Red List of Threatened Species [1]. As an icon of the Yangtze ecosystem [2] and the only freshwater porpoise in the world, this species is currently found only in the main stem of the middle and lower reaches of the Yangtze River and the adjoining Poyang and Dongting Lakes [3], [4]. Unfortunately, many of these porpoises live in areas that are undergoing rapid and increasing habitat destruction and suffering from sharply declining fish stocks. Consequently, these porpoise are at risk of extinction similar to the Yangtze River dolphin (*Lipotes vexillifer*), which was likely driven to extinction by human activity [1], [5].

Observing free-ranging finless porpoises presents a logistical challenge, as these animals take swift surface breaths, lack a dorsal fin, are small in size and have barely visible natural markings. Indeed, these animals are among the most difficult cetacean species to observe visually [6], [7].

Toothed whales (Odontoceti) rely primarily on their sophisticated acoustic sensing biosonar for communication, navigation and foraging [8]. The Yangtze finless porpoise uses sonar in an almost continuous manner (sound pressure level (SPL) of over 148 dB peak-to-peak re 1 µPa with an average inter-click-bout interval of 6.4 s) [9], which allows for acoustic monitoring of these animals.

Passive acoustic monitoring (PAM) is a rapidly evolving discipline that facilitates the investigation of the behavior of many aquatic mammals over a range of spatial and temporal scales (for reviews, see [10]), including at night and during adverse weather conditions. Additionally, this method enables round-the-clock monitoring for extended periods of time, which is logistically impossible for visual observations. Over the past decade, our understanding of porpoise behavior and distribution has improved greatly through the use of PAM [11]–[16].

Harbors and ports are common along the Yangtze River, and the structures of some of the platforms and installations extend down through the water column. These structures can attract large accumulations of plankton via illumination and hydrodynamic effects [17], provide shelter for other underwater organisms from currents or predators [18], [19] and influence local biodiversity and ecosystems. The results from acoustic surveys from the Yangtze Freshwater Dolphin Expedition of 2012 suggest that finless porpoises typically occupy the port areas at night for prolonged periods and frequently emit buzz sounds (short-range sonar sounds with minimum inter-click intervals shorter than 10 ms) [14]. In acoustic studies of dolphins, buzz sounds have been used as indicators of attempts at prey capture [20], feeding activity [7], [21] or foraging success [22], [23], and Yangtze finless porpoises also emit buzzes during prey capture dives [24]. The detection of nocturnal buzz sounds suggests that finless porpoises feed near the port area at night [14]. Mounting evidence from studies of circadian rhythms has shown that a wide variety of taxa tend to switch from nocturnal to diurnal activity patterns to adapt to metabolic demands (for a review, see [25]) and/or their physical and social environment (see [26] for a review). However, with the exception of information collected from a buoy-based PAM system near the outlet of Poyang Lake into the Yangtze River [27], little is known about the diel patterns of porpoise distributions. These shortcomings should be addressed, particularly with regard to the diel rhythms of finless porpoises in the vicinity of the port area, because the area is frequently subjected to artificial activities. Knowledge of the time-specific habitat preferences of these animals can not only help us identify ways to mitigate possible human interference, especially during times of peak porpoise presence, but also inform present and future conservation needs for this species, which is facing significant extinction pressure.

Because regional studies can often provide useful insights into broader patterns, the primary goal of this study was to describe the potential diel patterns of porpoises in port areas. Moreover, earlier findings indicate that Yangtze finless porpoises tend to frequent habitats with high densities of fish [28]; the rate of porpoise detection was significantly higher in areas with fish than in areas without fish [12]. Additionally, the significant negative correlation between the presence of Yangtze finless porpoises and the presence of ships suggests that porpoises tend to avoid boat traffic [11], and indirect and direct evidence of the tendency to avoid passing vessels has also been documented [13], [14]. We tested the hypothesis that prey availability triggers porpoise feeding and that boat traffic disrupts feeding. Porpoise movement patterns relative to the port were also analyzed.

## Methods

### Data Collection

#### Porpoise acoustic data

**Figure 1:**
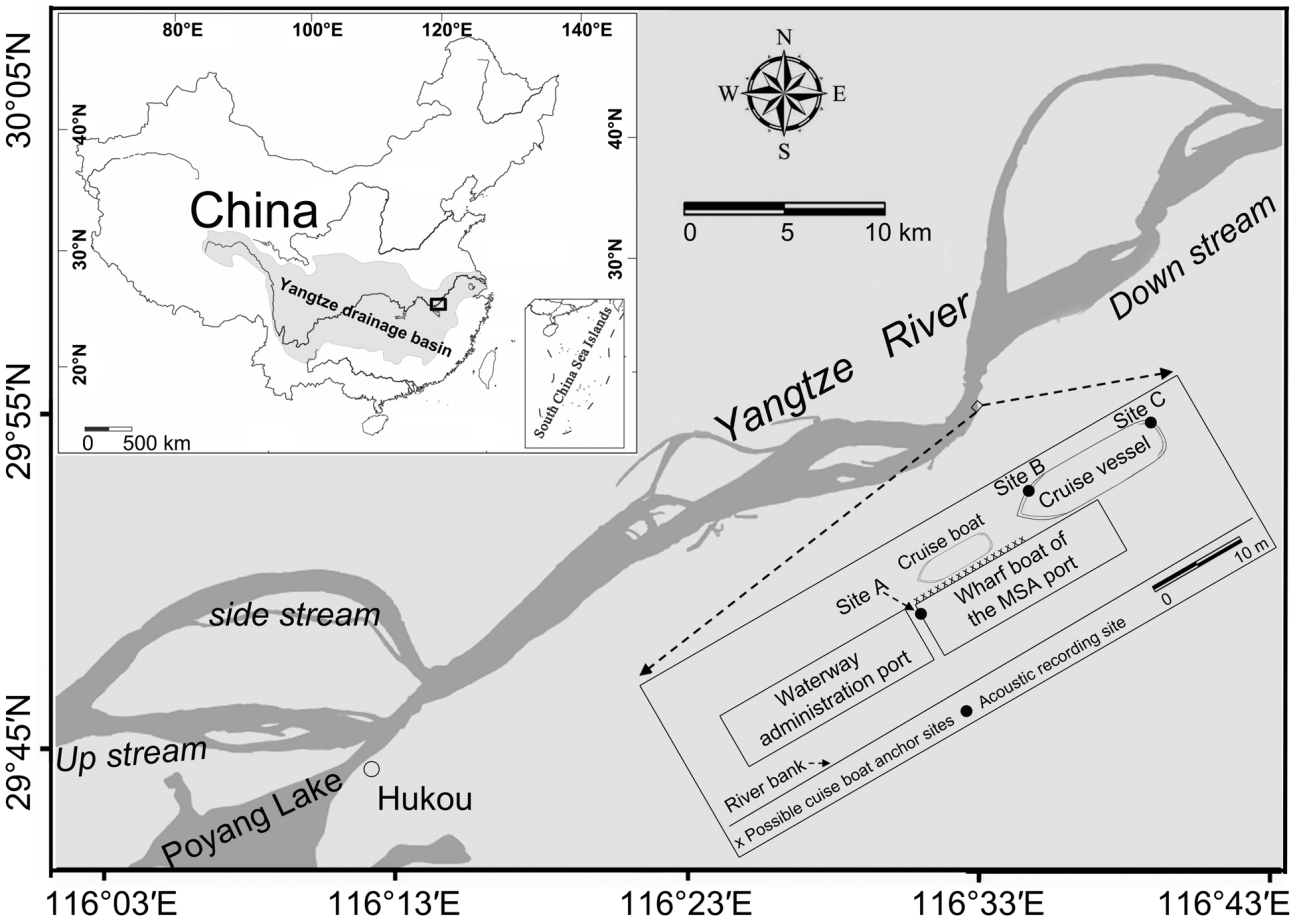
Map of the static acoustic monitoring area. The MSA port consisted of an anchored cruise vessel (27 m long and 5 m wide) and a rectangular wharf boat (39 m long and 9 m wide) connected to a waterway administration port of the same size. Three miniature stereo pulse event data loggers were deployed at two sites on the cruise vessel (sites A and B) and at one site on the wharf boat at the MSA port (site C). The possible cruise boat anchor sites represent possible sites for fish data monitoring. The inset map in the upper left corner shows the position of the study area in the middle region of the Yangtze drainage basin.

PAM of porpoise sonar sounds was conducted at the Pengze Maritime Safety Administration (MSA) Port (see Fig. 1) from 11∶00 am on 8 June to 7∶20 am on 23 June 2013. The MSA port consists of an anchored cruise vessel (27 m long and 5 m wide) and a rectangular wharf boat (39 m long and 9 m wide) connected to a waterway administration port of the same size. Each of these structures is water-based and arranged parallel to the south bank of the Yangtze River, approximately 5 m apart; no other water-based structures were located within 2 km of the area (Fig. 1). Three miniature stereo pulse event data loggers (A-tag; see details under “Porpoise sonar monitoring system” below) were used. Each data logger was tightly fastened to a stainless steel pole with plastic bands and deployed vertically at two sites on the cruise vessel (site A, 29°54′24″ N, 116°32′50″ E; site B, 29°54′25″ N, 116°32′51″ E) and at one site on the wharf boat (site C, 29°54′26″ N, 116°32′51″ E) (Fig. 1) with the hydrophone array of the A-tag deployed parallel to the current. The depth of the recording site ranged from 7 to 8 m, and each A-tag was deployed at a depth of 1.5 m depth (approximately 1 m deeper than the submerged part of the port) to minimize the possibility of obstructions to the sound.

### Fish Data

A maritime safety cruise boat-mounted single-beam Raymarine HD Digital Fishfinder (model C80, Raymarine UK Ltd., Fareham, UK) was used to log data while anchored at the port. The transducers of this echosounder can transmit 50 kHz as a narrow beam. However, to minimize the possible interference of the transmitted sound with the A-tags, the fish data were randomly sampled as echograms by photographing the monitoring screen of the Fishfinder at different diel phases (see below) from the 19^th^ to the 23^rd^ of June instead of being continuously monitored. The cruise boat was anchored along the wharf boat after each day’s expedition to a random site (Fig. 1), which resulted in fish data from multiple sites rather than one fixed point to represent the density of fish at the port.

#### Boat traffic data

The diel pattern of boats upriver and downriver of the study site was either visually counted or photographed with a Pentax camera (model Optio W60, PENTAX Corporation, Tokyo, Japan) shooting at 1 min intervals from the 15^th^ to the 23^rd^ of June. The boat’s safety lights made photography possible during the night. The camera was fixed at an advantageous location on the second deck of the cruise vessel (Fig. 1) and oriented to capture every vessel that passed during 1-min periods. As demonstrated by a preliminary study, the 1-min setting is sufficient to capture every passing vessel except for speed boats, which are rare in this region.

### Ethical Statement

The research was carried out under a research permit issued to the Institute of Hydrobiology of the Chinese Academy of Sciences by the Ministry of Agriculture of China. The approval number from the Fishery Bureau of the General Office of the Ministry of Agriculture was 2012–114.

### Porpoise Sonar Monitoring System

Two types of A-tags (including towed and fixed types) were used in this study. A brief description of these autonomous acoustic recorders is provided here, and a detailed description is provided by Akamatsu et al. (2005) [29]. Each A-tag (ML200-AS2, Marine Micro Technology, Saitama, Japan) consisted of two ultrasonic hydrophones, a band-pass filter (−3 dB with a 55–235 kHz range) and a high-gain amplifier (+60 dB). The hydrophones (model MHP-140ST; Marine Micro Technology, Saitama, Japan) had the following parameters: sensitivity at −201 dB re 1 V/µPa at 1 m distance and a frequency response range from 100 to 160 kHz within 5 dB with the primary and secondary hydrophones separated at a fixed range of either 195 mm (towed type) or 590 mm (fixed type) to form a stereo hydrophone array. The data logger had directional sensitivity with 0° on-axis and 90° off-axis detection thresholds of 129 dB and 140 dB peak-to-peak (reference pressure 1 µPa at 1 m), respectively [30], [31]. This setup is considered suitable for monitoring the presence of porpoises, as their apparent source levels are 163.7–185.6 dB peak-to-peak re 1 µPa [32]; the source levels of the 90° and 120° off-axis sonar signals were above the 160 dB peak-to-peak re 1 µPa [30], [33]. Instead of recording the raw waveforms, the system registered ultrasonic pulse events. The peak sound pressure of each pulse, the absolute time of sound detection and the difference in arrival time (TD) of the same pulse at the two ultrasonic hydrophones were recorded up to 2000 (towed type) or 500 (fixed type) times per s. The dynamic range of the data logger was 129 dB to 160 dB peak-to-peak re 1 µPa with an internal thermal noise of approximately 134 dB, which was selected as the hardware detection threshold level. Once the primary hydrophone was triggered by a pulse sound that surpassed the preset hardware detection threshold level, the 10-bit (1024 count), high-speed counter would wait for the trigger at the other hydrophone and measure the TD between the stereo hydrophones at a resolution of 271 ns (towed type) or 1084 ns (fixed type). Each A-tag was equipped with a 128 MB flash memory card and a commercially available lithium battery (CR2) (towed type) or two UM-1 batteries (LR20) (fixed type). Depending on porpoise echolocation activity and background noise, this setup generally ensured autonomous monitoring operations for 40–60 h (towed type) or 1 month (fixed type) with a time drift of less than 1 s per day [29]. To ensure continuous recording, the three A-tags were never retrieved simultaneously for battery exchange. Theory and experimental evidence [15], [16] suggest that the effective detection distance for porpoises is 300 m.

### Diel Classification

The 24 h of the day were divided into five diel phases (night1, morning, day, evening, night2) following the method proposed by Todd et al. (2009) [7]. The time points used to divide these diel phases were as follows: between night1 and morning = civil twilight start; between morning and day = 2*sunrise-civil twilight start; between day and evening = 2*sunset-civil twilight end; between evening and night2 =  civil twilight end; between night1 and night2 = 12∶00 pm. Sunrise and sunset referred to the times when the upper edge of the sun was on the horizon. Civil twilight started in the morning and ended in the evening when the center of the sun was geometrically 6° below the horizon. These data were obtained from the web site of the US Naval Observatory (http://aa.usno.navy.mil). The categories of night1 and night2 were combined to make up the rest of the diel phases of the night and reserved for further analysis.

### Data Analysis

During the off-line signal processing of the A-tag data, two-step procedures were applied using Igor Pro 5.01 software (Wave Metrics, Lake Oswego, OR, USA) to filter out the non-porpoise clicks and extract the porpoise sonar. A custom-made, multi-parameter filter program was used during the pilot step to classify the recorded pulses as originating from porpoises, boat traffic or other sources (for details, see [14]). Click trains were defined as sets of five clicks, and bouts consisting of fewer than five clicks were discarded [34], [35]. During the second step, the number of false detections was further reduced by double-checking the detector output. We pooled all of the data from the three A-tags; if a signal was detected by more than one A-tag, only one was randomly selected to avoid over sampling. An echolocation encounter was defined as a series of click trains separated by eight min or less. Thus, if bouts of porpoise sonar were separated by more than eight min, they were considered to come from distinct echolocation encounters.

**Figure 2:**
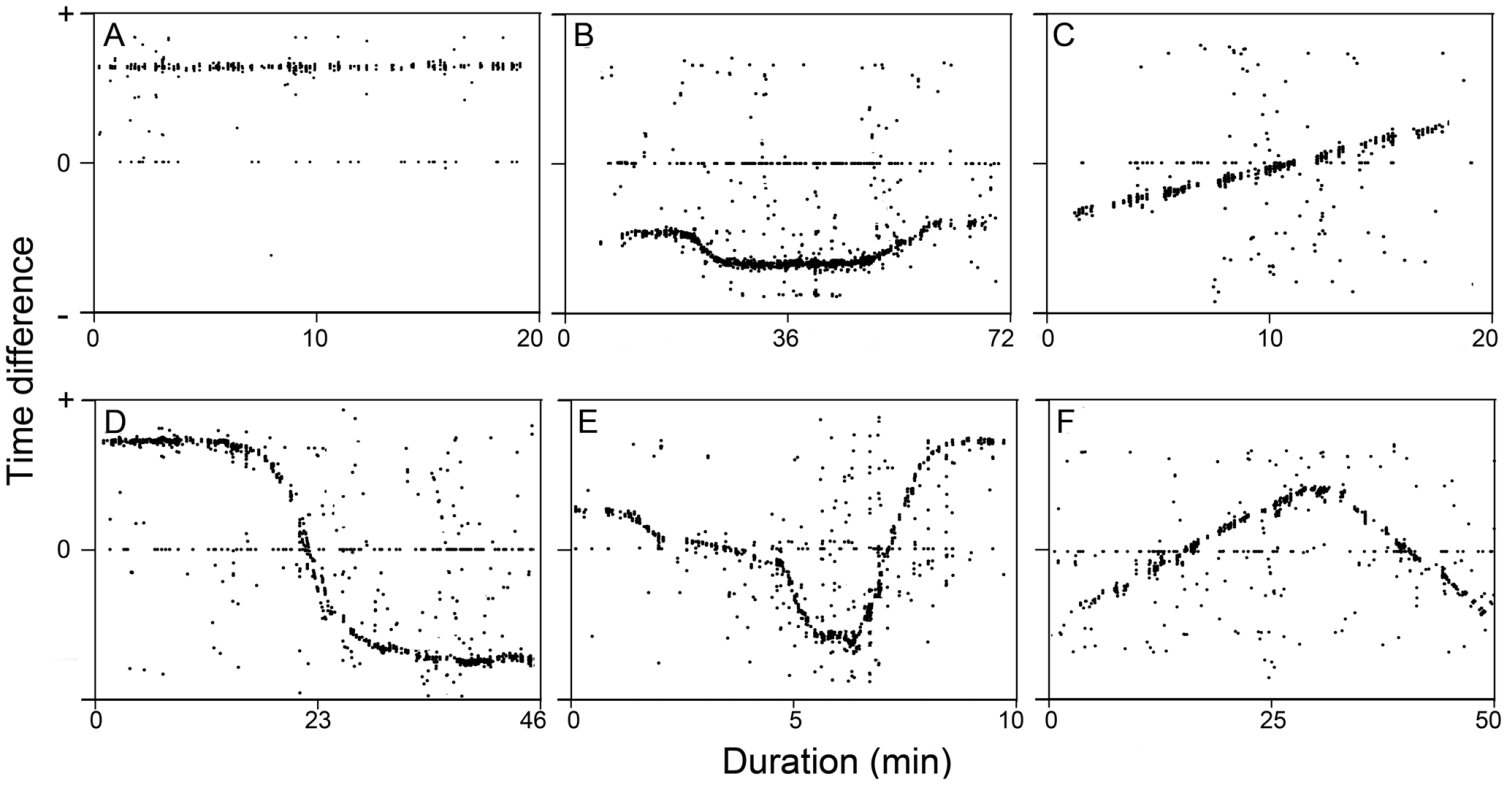
Types of trajectory. The positive and negative time differences indicate that the sound came from upstream or downstream, respectively. A value of zero denotes that the sound came from a point equidistant from the two ultrasonic hydrophones of the A-tag (the passing point). A = upstream stay, B = downstream stay, C = straight up, D = straight down, E = milling and up, F = milling and down.

Because the A-tag hydrophone array was deployed parallel to the water current and the primary hydrophone was directed downstream, the recorded TD was assigned a value of zero if the sound came from a point midway between the two ultrasonic hydrophones (the passing point). In contrast, a positive or a negative value was recorded if the source was upstream or downstream (with the A-tag set as the reference point), respectively. The gradual changes in the TD of the sonar detected within each echolocation encounter indicated the underwater movement of the porpoise (acoustic trajectory). A trajectory lasting more than 20 s with at least three click trains was categorized into one of the following classes: upstream stay (staying upstream without a passing point, Fig. 2A), downstream stay (staying downstream without a passing point, Fig. 2B), straight up (moving from downstream to upstream with one passing point, Fig. 2C), straight down (moving from upstream to downstream with one passing point, Fig. 2D), milling and up (moving upstream after moving back and forth with at least two passing points, Fig. 2E) and milling and down (moving downstream after moving back and forth with at least two passing points, Fig. 2F). In some cases, more than one trajectory (multi-trajectories) was observed within an echolocation encounter, which facilitated porpoise counting [15]. Only the longest trajectory was counted if the multi-trajectories were indistinguishable at either the beginning and/or ending time during the analysis of trajectory duration.

For the analysis of fish resources, individual echo trace counting was performed. Individual echo traces with a target strength over −60 dB were counted, which corresponds to the approximate size of a fish with a swim bladder at 2 cm [36]. The finless porpoise in Hong Kong waters can feed on fish ranging from 5.5 cm to 58.6 cm [37], and the typical body length of the favorite prey of captive Yangtze finless porpoises is 7 cm (Wang CQ, personal communication), which is well above the selected target strength. Note that the beam pattern is quite narrow at 8° and transmits as a corn-shaped sound beam. We excluded echo traces shallower than 0.5 m because the beam width did not cover the entire 7 cm body length of the typical prey. Fish density was calculated as the number of individual echo traces in each echogram divided by the observed volume of the echosounder beam. During observation, the boat did not move to enable the clear echo traces of each fish to be obtained for a relatively longer duration than an observation from a moving vessel. We did not sort the echo traces by target strength because we did not measure the attack angle of the acoustic beam to the fish.

Porpoise acoustic detection data (click trains and buzzes), fish density in the port area and boat traffic (including upriver boats, downriver boats and the combination of both) for each day were first assigned to different diel phases (night1, morning, day, evening, night2 and night), and the data from each diel phase were further grouped into 10 min bins to account for the difference in sampling effort across diel phases. Echolocation encounter and trajectory were also assigned to different diel phases. During counting, echolocation encounters and trajectories that crossed boundaries between diel phases were assigned to the dominant phase that contained the greater portion. For the analysis of diel pattern, echolocation encounters and trajectories that spanned multiple days were segmented, and each segment was assigned an appropriate diel phase.

### Statistical Analysis

The Kolmogorov–Smirnov test and Levene’s test for equality of variances were used to analyze the distribution of the data and the homogeneity of the variance, respectively. Nonparametric statistical analyses [38] were used because exploratory analysis showed that the data were not normally distributed (Kolmogorov-Smirnov test: *p*<0.05). The Kruskal–Wallis nonparametric test [38] was used to examine the overall difference across diel phases for all of the above-listed parameters. Duncan’s multiple comparison test [38] was used for all post hoc comparisons to determine how these parameters varied with diel phase. Diel pattern analysis was conducted for 5 phases (night1, morning, day, evening and night2) and 4 phases (morning, day, evening and night). Spearman’s rank order correlation [38] was employed to investigate the correlation among porpoise acoustics, boat traffic and fish density. For the correlation between porpoise acoustics and boat traffic, porpoise acoustics recorded simultaneously with boat traffic monitoring (from the 15^th^ to the 23^rd^ of June) were screened out and matched in the form of 10 min bins. As for the correlation between porpoise acoustics and fish density, the relevant data from the 19^th^ to the 23^rd^ of June were grouped and calculated for the 5 diel phase pattern. The Mann–Whitney *U*-test [38] was used to further analyze the presence or absence of clicks or buzzes with patterns of boat traffic. Statistical analyses were performed using Statistical Package for the Social Sciences (SPSS) 16.0 for Windows (SPSS Inc., Chicago, U.S.A.). Significance was accepted at *p*<0.05.

## Results

**Figure 3:**
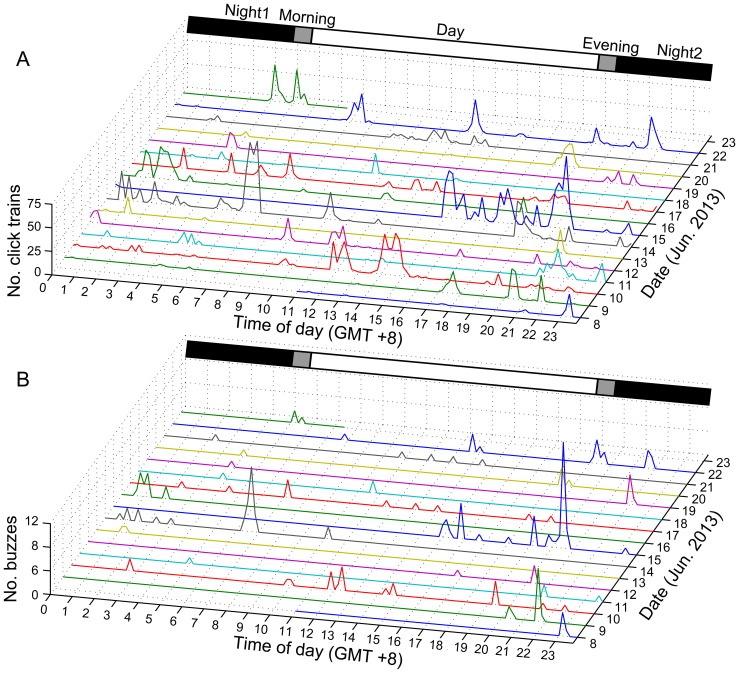
The daily occurrence of porpoise click trains (A) and buzzes (B) by diel phase and time of day. The data were grouped into 10(hour GMT +8).

Porpoise sonar signals (both clicks and buzzes) were monitored every day (Fig. 3) for a total of 21,380 min of acoustic monitoring time; of that total, 1233 min (5.77% of the total recording time) contained porpoise sonar. A combined total of 3372 click trains were recorded, 190 (5.63%) of which were buzzes (Table 1). The average duration of the 168 echolocation encounters was 10.73±1.25 min (mean ± standard error, SE), with a maximum duration of 98.50 min (Table 1); 150 (89.3%) of these encounters involved one animal, 14 encounters involved two porpoises, and three encounters (1.8%) and one (0.6%) encounter involved three and four porpoises, respectively.

**Table 1:** Descriptive statistics of porpoise acoustic detection (including minutes of clicks and number of click trains and buzzes) and echolocation encounters (including number, group size and duration) for each day.

Date	Minutes of clicks	No. of click trains	No. of buzzes	Echolocation encounters			
				N	Group size	Duration (min)	
						mean±SE	Range
6/8/2013	17	40	5	7	1	2.83±1.82	0.002–9.95
6/9/2013	58	155	13	9	1	10.00±4.79	0.001–38.51
6/10/2013	146	411	21	24	1–3	8.55±4.26	0.002–95.80
6/11/2013	96	187	4	10	1–2	13.02±7.22	0.002–76.67
6/12/2013	72	151	4	11	1	9.13±3.54	0.006–36.49
6/13/2013	24	55	2	5	1–3	7.55±4.34	0.007–23.38
6/14/2013	202	624	30	21	1–2	13.65±3.86	0.001–51.80
6/15/2013	178	654	47	10	1–4	25.78±7.75	1.437–61.23
6/16/2013	83	240	13	10	1–2	12.43±5.22	0.004–49.67
6/17/2013	86	201	10	17	1	6.65±1.60	0.001–23.56
6/18/2013	19	39	3	6	1	4.24±1.55	0.002–8.88
6/19/2013	30	69	7	4	1	14.13±3.39	8.083–20.65
6/20/2013	36	94	5	4	1–2	14.57±13.35	0.002–54.53
6/21/2013	56	94	5	13	1	6.67±2.32	0.008–24.69
6/22/2013	90	240	18	15	1	9.28±3.55	0.001–34.16
6/23/2013	40	118	3	2	1	35.30±1.95	33.35–37.26
Mean±SE	77.06±14.01	210.75±48.09	11.88±3.05	10.50±1.25		10.73±1.25	0.001–95.80
sum	1233	3372	190	168			

The duration of echolocation encounters is given as the mean ± standard error (SE) and range.

### Trajectory Types

Of the 18 multi-trajectory echolocation encounters, six involved two unambiguous multi-trajectories, resulting in a combined total of 174 measured trajectories (Table 2). One hundred and nine trajectories could be classified: nine (8.26%) and 14 (12.84%) were upstream stay and downstream stay, respectively; 30 (27.52%) and 35 (32.11%) were straight up and straight down, respectively; and 19 (17.43%) and two (1.83%) were milling and up and milling and down, respectively (Table 2).

**Table 2:** Descriptive statistics of the different trajectory types.

	Upstream stay	Downstream stay	Straight up	Straight down	Milling and up	Milling and down	Unidentified	All
Mean±SE	4.45±1.09	19.85±5.69	13.54±2.13	11.17±2.83	32.55±4.45	15.14±4.89	0.96±0.25	10.49±1.20
Min−Max	0.50–9.83	0.64–74.01	1.37–49.67	0.94–95.80	4.22–61.23	10.25–20.03	0.0001–8.68	0.0001–95.80
N	9	14	30	35	19	2	65	174

The duration of each type of trajectory (in min) is given as the mean ± standard error (SE) with minimum (Min) and maximum (Max) values.

Significant differences in the duration of the different trajectory types were observed (Kruskal-Wallis χ2 = 28.17, df = 6, *p*<0.01). Specifically, the duration of the milling and up trajectory was significantly longer than the straight down trajectory (Duncan’s multiple-comparison test; *p*<0.05), and the upstream stay was significantly shorter than the milling and up (Duncan’s multiple-comparison test; *p*<0.05) (Table 2).

### Diel Pattern of Biosonar Behavior and the Movement of Porpoises

**Figure 4:**
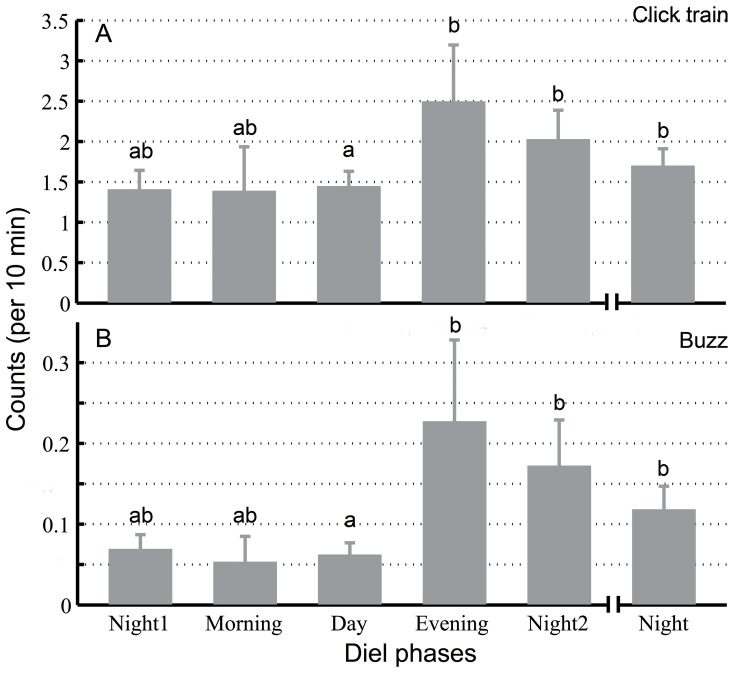
Diel pattern of the frequency of click trains (A) and buzzes (B) per 10 min. The data were combined and averaged across the entire acoustic monitoring period. Error bars (mean ± SE) with different lowercase letters refer to post hoc Duncan’s multiple-comparison tests that yielded significant results (*p*<0.05).

Significant differences in the frequency of porpoise click trains (per 10 min) were observed among the diel patterns (5 phases: Kruskal-Wallis χ2 = 21.16, df = 4, *p*<0.01; 4 phases: Kruskal-Wallis χ2 = 19.62, df = 3, *p*<0.01). In particular, the frequency of click trains was significantly lower during the day (mean ± SE: 1.44±0.19 per 10 min) than during the evening (mean ± SE: 2.49±0.70 per 10 min), night2 (mean ± SE: 2.03±0.36 per 10 min) and night (mean ± SE: 1.69±0.21 per 10 min) (Duncan’s multiple-comparison test; *p*<0.05) (Fig. 4A). The frequency of porpoise buzzes was significantly different among diel phases (5 phases: Kruskal-Wallis χ2 = 13.66, df = 4, *p*<0.01; 4 phases: Kruskal-Wallis χ2 = 12.32, df = 3, *p*<0.01). Buzz frequency was significantly lower during the day (mean ± SE: 0.06±0.02 per 10 min) than in the evening (mean ± SE: 0.23±0.10 per 10 min), night2 (mean ± SE: 0.17±0.06 per 10 min) and night (mean ± SE: 0.12±0.03 per 10 min) (Duncan’s multiple-comparison test; *p*<0.05) (Fig. 4B).

**Figure 5:**
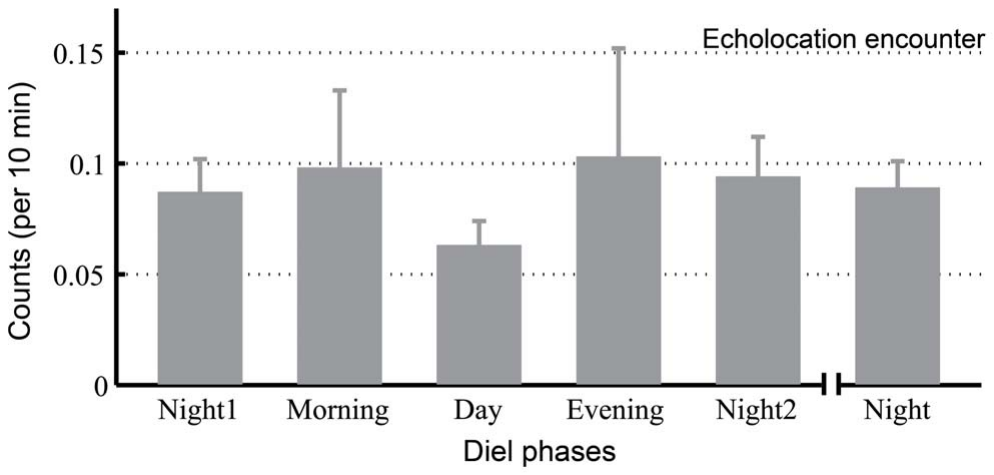
Diel pattern of echolocation encounters. The data were combined and averaged across the entire acoustic monitoring period.

No significant diel patterns were observed in either the frequency of echolocation encounters (5 phases: Kruskal-Wallis χ2 = 4.62, df = 4, *p* = 0.33; 4 phases: Kruskal-Wallis χ2 = 5.39, df = 3, *p* = 0.15) (Fig. 5) or the duration of the echolocation encounters (5 phases: Kruskal-Wallis χ2 = 3.03, df = 4, *p* = 0.55; 4 phases: Kruskal-Wallis χ2 = 2.23, df = 3, *p* = 0.0.53) (Table 3).

**Table 3:** Descriptive statistics of the diel pattern of echolocation encounter duration.

	Night1	Morning	Day	Evening	Night2	Night
Mean±SE	9.25±2.08	12.53±5.36	10.87±2.03	10.53±3.67	10.77±2.62	10.02±1.67
Min−Max	0.001–49.67	0.01–39.64	0.01–95.80	0.001–41.80	0.001–76.67	0.001–76.67
N	37	8	76	11	38	75

The duration (in min) is given as the mean ± standard error (SE) with minimum (Min) and maximum (Max) values.

**Figure 6:**
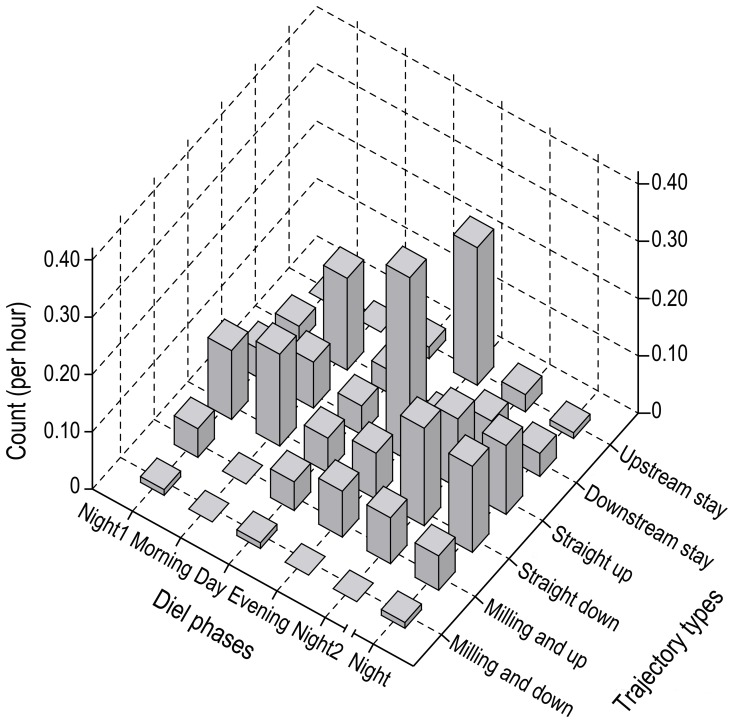
Frequency of different types of trajectory by diel phase. To calculate the frequency of occurrence of each trajectory type, all of the trajectories recorded in each diel phase were first classified by type and then divided by the total recording time for each diel phase.

The stay trajectory types peaked in the morning and evening. The upstream stay, with a frequency of 0.24 counts per h (Fig. 6), accounted for 33% of the trajectories detected in the evening. The downstream stay, with a frequency of 0.16 counts per h (Fig. 6), accounted for 40% of the trajectories detected in the morning. The straight moving type also peaked in the morning and evening. The straight up type, with a frequency of 0.32 counts per h (Fig. 6), accounted for 44% of the trajectories identified in the evening, and the straight down type, with a frequency of 0.16 counts per h (Fig. 6), accounted for 40% of the identified trajectories in the morning. The milling trajectories were observed less often. The milling and up trajectory peaked in the evening and night2, with a frequency of 0.08 counts per h. The maximum occurrence rate of the milling and down trajectory was 0.01 counts per h, and no milling trajectories were observed in the morning (Fig. 6).

### Diel Pattern of Fish Density

**Figure 7:**
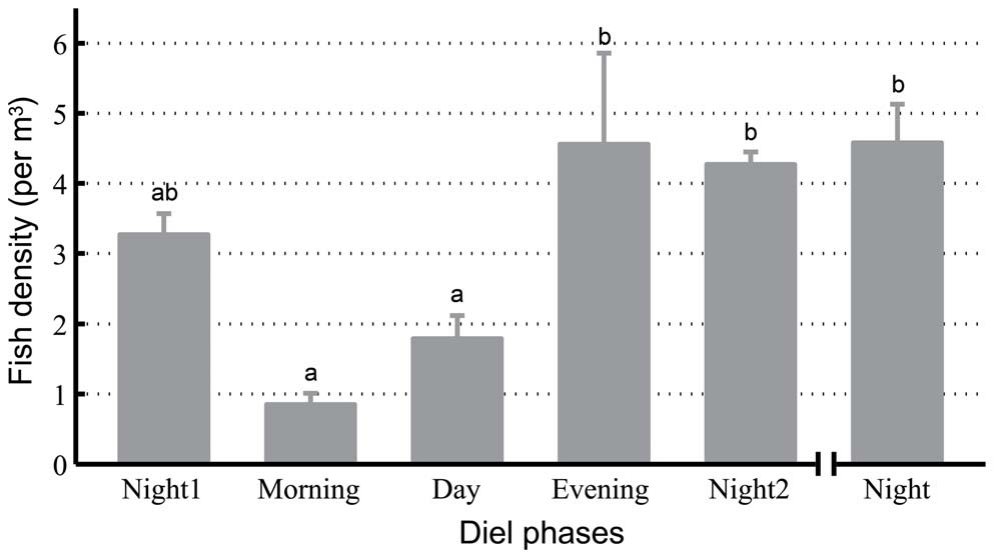
Diel pattern of fish density in the port areas. The data were combined and averaged across the entire echosounder monitoring period. Error bars (mean ± SE) with different lowercase letters refer to post hoc Duncan’s multiple-comparison tests that yielded significant results (*p*<0.05).

Significant diel patterns in fish density were observed in the port area (5 phases: Kruskal-Wallis χ2 = 10.06, df = 4, P = 0.04; 4 phases: Kruskal-Wallis χ2 = 10.16, df = 3, *p* = 0.02). Specifically, fish densities in the morning (mean ± SE: 0.85±0.16 fish per m^3^) and during the day (mean ± SE: 1.79±0.33) were significantly lower than fish densities in the evening (mean ± SE: 4.56±1.30), at night2 (mean ± SE: 4.27±0.18) and at night (mean ± SE: 4.58±0.55) (Duncan’s multiple-comparison test; *p*<0.05) (Fig. 7).

### Diel Pattern of Boat Traffic

**Figure 8:**
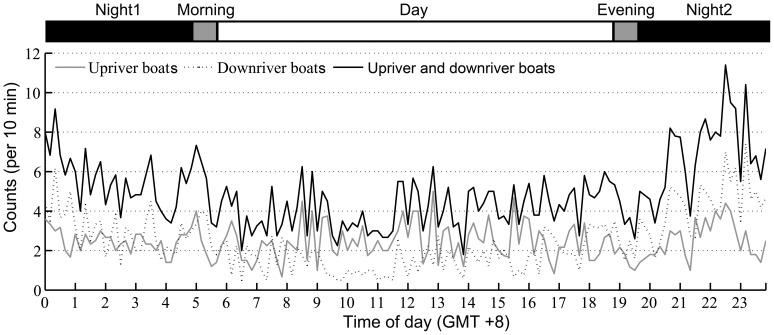
Frequency of boat traffic (including upriver boats, downriver boats and the combination of upriver and downriver boats) by diel phase and time of day. The data were pooled and averaged over the entire boat monitoring period. The horizontal bar at the top indicates the diel phase, and the bar at the bottom denotes the time of day (hour GMT +8).

**Figure 9:**
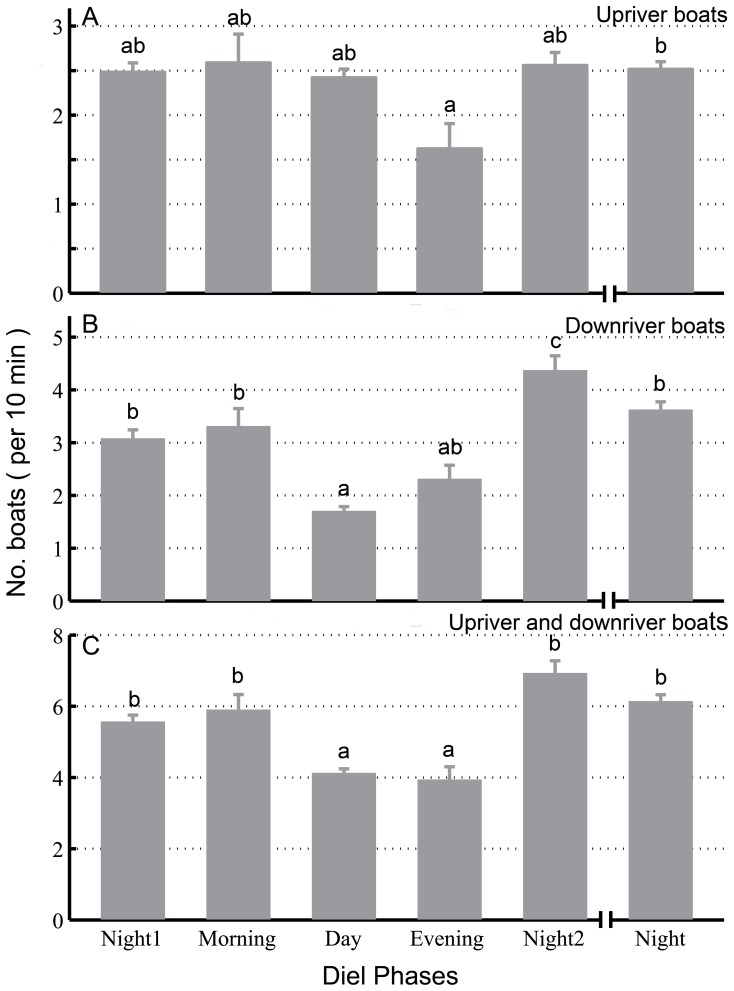
Diel pattern of boat traffic (upriver boats in A, downriver boats in B, upriver and downriver boats in C). The data were combined and averaged over the entire boat monitoring period. Error bars (mean ± SE) with different lowercase letters refer to post hoc Duncan’s multiple-comparison tests that yielded significant results (*p*<0.05).

Significant diel patterns were observed in the frequency of boats passing upriver (5 phases: Kruskal-Wallis χ2 = 10.28, df = 4, *p* = 0.04; 4 phases: Kruskal-Wallis χ2 = 10.28, df = 3, *p* = 0.02) (Figs. 8 and 9A). In particular, the frequency was significantly lower in the evening (mean ± SE: 1.62±0.28 count per 10 min) than at night (mean ± SE: 2.51±0.09) (Duncan’s multiple-comparison test; *p*<0.05) (Fig. 9A). The frequency of the passage of boats downriver was significantly different among diel phases (5 phases: Kruskal-Wallis χ2 = 133.59, df = 4, *p*<0.01; 4 phases: Kruskal-Wallis χ2 = 122.56, df = 3, *p*<0.01) (Figs. 8 and 9B). In particular, the frequency of boats passing downriver during the day (mean ± SE: 1.70±0.09) was significantly lower than that of all of the other diel phases except the evening (mean ± SE: 2.31±0.26) (Duncan’s multiple-comparison test; *p*>0.05) (Fig. 9B). Significant diel patterns were observed in the frequency of passage of all types of boats (5 phases: Kruskal-Wallis χ2 = 86.45, df = 4, *p*<0.01; 4 phases: Kruskal-Wallis χ2 = 81.41, df = 3, *p*<0.01) (Figs. 8 and 9C). In particular, the frequency of passage of all boats during the day (mean ± SE: 4.12±0.12) or in the evening (mean ± SE: 3.93±0.37) was significantly lower than that of the other diel phases (Duncan’s multiple-comparison test; *p*<0.05); no significant differences in frequency were observed among these other diel phases or between the day and the evening (Duncan’s multiple-comparison test; *p*>0.05) (Fig. 9C).

### Correlation among Porpoise Sonar, Fish Density and Boat Traffic

Twenty-two pairs of matched porpoise acoustic and fish density data were derived (including the 5 diel phases from the 19^th^ to the 22^nd^ plus night1 and the morning of the 23^rd^ of June). Significant correlations were observed between fish density and the frequency of porpoise click trains (Spearman’s rho = 0.771, *p* = 0.04, n = 22) and between fish density and the frequency of porpoise buzzes (Spearman’s rho = 0.829, *p* = 0.03, n = 22) (Table 4).

**Table 4:** Correlation among dolphin acoustic, fish density and boat traffic.

		Fish density	Boat traffic		
			Upriver boat	Downriver boat	All boats
Porpoise acoustic	Click train	*	n	n	n
	buzz	*	n	n	n

*Represents significance at p<0.05; n represents not significant with p>0.05.

A combined total of 682 simultaneously matched pairs of dolphin acoustic and boat traffic data (10 min bins) were obtained. The number of click trains was not significantly correlated with the passage of boats upriver, downriver or both (Spearman’s rho = −0.016, *p* = 0.684, n = 682, Spearman’s rho = −0.045, *p* = 0.241, n = 682 and Spearman’s rho = 0.012, *p* = 0.755, n = 682, respectively) (Table 4). Furthermore, no significant differences were observed in the presence or absence of click trains with the frequency of the passage of boats upriver, downriver or both (Mann-Whitney *U*-test; z = −1.455, df = 682, *p* = 0.146; Mann-Whitney *U*-test; z = −0.188, df = 682, *p* = 0.851 and Mann-Whitney *U*-test; z = −1.161, df = 682, *p* = 0.245, two-tailed, respectively). There was also no significant association between the number of porpoise buzzes and the passage of boats upriver (Spearman’s rho = −0.056, *p* = 0.146, n = 682), downriver (Spearman’s rho = −0.008, *p* = 0.837, n = 682) or both (Spearman’s rho = −0.044, *p* = 0.251, n = 682) (Table 4). No significant differences in the presence or absence of buzzes were observed with the frequency of the passage of boats upriver, downriver or both (Mann-Whitney *U*-test; z = −0.342, df = 682, *p* = 0.732; Mann-Whitney *U*-test; z = −1.145, df = 682, *p* = 0.252 and Mann-Whitney *U*-test; z = −0.328, df = 682, *p* = 0.743, two-tailed, respectively).

## Discussion

Passive acoustic monitoring showed that Yangtze finless porpoises occupy port areas (Fig. 2) more routinely as described by Wang et al. (2014). The majority of the echolocation encounters involved single animals, indicating that porpoises tend to be solitary.

### Correlation between Porpoise Biosonar Behavior and Fish Density

Distinct diel patterns in porpoise acoustics were observed, with significantly more click trains and buzzes detected in the evening and at night than during the day. These findings suggest that porpoises are primarily engaged in crepuscular and nocturnal foraging in the port area. Considering that no significant diel patterns were observed in the frequency and duration of porpoise echolocation encounters, the diel pattern of porpoise acoustic behavior appears to be due to porpoises producing more click trains and buzzes at night [39] and possibly in the evening. These findings are consistent with findings from previous studies that show no reduced nocturnal activity among Yangtze finless porpoises in captivity [6]. The index of stomach contents of finless porpoises in Japan was also higher at night than during the day, with peak values observed at night [40].

Mounting evidence indicates that some marine mammals tend to feed at night. For example, Dall’s porpoises (*Phocoenoides dalli*) are thought to forage mainly at night and/or at dawn, as greater amounts of stomach content were observed in animals captured in the morning [41]. Pantropical spotted dolphins (*Stenella attenuata*) have been shown to be primarily nocturnal and crepuscular feeders [42]–[44]. The feeding buzz rates and/or foraging-related behavior of wild harbor porpoises (*Phocoena phocoena*) [7], [21], [45], Cuvier’s beaked whales (*Ziphius cavirostris*), Blainville’s beaked whales (*Mesoplodon densirostris*) [46], Ganges River dolphins (*Platanista gangetica*) [34], [47], Risso’s dolphins (*Grampus griseus*) [48] and deep-diving echolocating odontocetes off of the Hawaiian islands [35] were higher at night than during the day. Free-ranging white-beaked dolphins (*Lagenorhynchus albirostris*) have been shown to forage only at night when monitored with bio-logging tags and passive acoustic methods [49].

In this study, porpoise biosonar behavior was significantly correlated with fish density, which supports previous findings that significantly more finless porpoises are encountered in areas with fish than in areas without fish [12]. Moreover, the higher fish densities observed in the evenings and at night in this study are consistent with more recent fixed echosounder detection findings that fish in the Yangtze River are more active (i.e., more fish are detected) and employ river bank-oriented movement at night [50].

The observation of no significant diel patterns in the frequency and duration of porpoise echolocation encounters indicates that the porpoises were always in the port area, even during the daytime, which highlights the importance of the port area.

### Diel Movement Patterns of Porpoises

Diel behavioral patterns have been correlated with spatio-temporal variations in food availability in common dolphins (*Delphinus capensis*) [51], [52], pantropical spotted dolphins (*S. attenuata*) [42], [43], spinner dolphins (*Stenella longirostris*) [53], dusky dolphins (*Lagenorhynchus obscures*) [54], striped dolphins (*Stenella coeruleoalba*) [55], harbor porpoises (*P. phocoena*) [7], Risso’s dolphins (*G. griseus*) [48], Heaviside’s dolphins (*Cephalorhynchus heavisidii*) [56], Ganges River dolphins (*P. gangetica*) [34] and Hector’s dolphins (*Cephalorhynchus hectori*) [57]. In this study, finless porpoises tended to migrate or stay downstream in the morning and then migrate or stay upstream in the evening. Milling and loop trajectories were possibly correlated with prey capture, and the lack of milling trajectories observed in the morning coincided with the lowest fish densities in the port. However, the milling trajectory peaked in the evening and night2, a phenomenon that was also observed in Ganges River dolphins [34], and the present study showed that the peak milling trajectory matched the peak fish density in the port. It is possible that these movements follow the local migration of prey, but further studies are needed to confirm this hypothesis.

### Correlation between Porpoise Sonar and Boat Traffic

The boat traffic observed in this study primarily consisted of large sand carriers and very few fishing boats. This pattern may be due to the prohibition on fishing in the Yangtze River from the first of April through the 30^th^ of June or to the nearby presence of the fishery law enforcement agency, which limits illegal fishing activities.

A broad range of organisms have been shown to exhibit diel foraging habits that are influenced by the trade-offs between foraging efficiency and predation risk [58]. In the Yangtze River system, however, finless porpoises are at the top of the food chain, which suggests that their diel foraging habits are not predicated on avoiding predators. The Yangtze River system has been severely affected by numerous types of anthropogenic activity, especially the high volume of boat traffic; it is likely that these activities are equivalent to predation risk. In this study, no significant correlation was observed between porpoise sonar and boat traffic, and no remarkable differences in boat traffic were observed in the presence or absence of porpoise acoustics (including click trains and buzzes). These findings suggest that prey availability in the port area are the primary factor that attracts porpoises. However, the lack of a significant correlation between porpoise acoustics and boat traffic does not rule out possible negative impacts of boat traffic on porpoise behavior. Indeed, indirect and direct evidence of finless porpoises avoiding passing vessels has been documented [13], [14]. We should not exclude the effect of anthropological noise on the presence of porpoises. The lack of a significant correlation between porpoise acoustics and boat traffic may be due to a forced feeding schedule because the Yangtze River is significantly overfished and porpoises are most likely forced to tolerate disturbances from boats in the port areas to find prey.

Porpoise acoustics (click trains and buzzes) peak in the evening, suggesting that this is the preferred time for porpoise feeding. This finding is consistent with the plateau in fish density (Figs. 4,7 and 9).

### Limitations

The number of echolocation encounters is not necessarily equal to the number of individual porpoises. Interference resulting from the movement of porpoises in and out of the detection range of the instrument and periods of silence during which the porpoises were in the vicinity of the A-tags may have resulted in duplicate acoustic detections of the same animal. Additionally, non-vocal animals, even those within range of the equipment, were not detected [59], which resulted in a conservative estimate of the numbers of individual porpoises.

## Conclusions

Porpoise sonar was frequently detected in the port areas. Recordings tended to consist of feeding buzzes, and a majority of echolocation encounters involved single animals, indicating that solitary porpoises were frequently present and feeding in the port area. The distinct diel patterns of porpoise biosonar behavior showed that the click trains and buzzes occurred much more frequently in the evening and at night than during the day, which suggests that porpoises in the region were primarily engaged in crepuscular and nocturnal foraging. Porpoise sonar was significantly correlated with fish density but not with boat traffic; it is possible that overfishing in the Yangtze River has forced the porpoises to tolerate the disturbances caused by boats in the port areas to capture prey. Overall, prey availability appears to be the primary factor attracting porpoises, and the evening, when porpoise acoustics peak, seems to represent the preferred period for porpoise feeding. The plateau in fish density and the decrease in boat traffic in the evening further support this preference. Additionally, porpoises tend to stay or migrate downstream in the morning and stay or migrate upstream in the evening; it is possible that they are following prey.

No studies have examined possible seasonal (for example, changes due to the increased metabolic burden imposed by pregnancy), yearly or site-specific variations in patterns of porpoise activity. Longer-term and wider-ranging monitoring at more locations is needed to identify and mitigate potential threats to this species.
